# Supplementary material for: Resolving the full spectrum of human genome variation using Linked-Reads
Source: Genome Res. 2019 Apr;29(4):635–45. doi: 10.1101/gr.234443.118 (PMC6442396; doi:10.1101/gr.234443.118)
Supplement: Supplemental Material [file supp_gr.234443.118_Supplemental_longranger-2.2.zip › longranger-2.2.tar.bz2!/lr2.2-public/lib/stan/cmdstan/stan/doc/api/html/agrad_2rev_2matrix_2log__sum__exp_8hpp_source.html]

Stan: src/stan/agrad/rev/matrix/log\_sum\_exp.hpp Source File


|  |  |
| --- | --- |
|  | Stan  2.5.0  probability, sampling & optimization |


- Main Page
- Related Pages
- Namespaces
- Classes
- Files

- File List
- File Members

All Classes Namespaces Files Functions Variables Typedefs Enumerations Enumerator Friends Macros Pages

- src
- stan
- agrad
- rev
- matrix

log\_sum\_exp.hpp

Go to the documentation of this file.

1 #ifndef STAN\_\_AGRAD\_\_REV\_\_MATRIX\_\_LOG\_SUM\_EXP\_HPP

2 #define STAN\_\_AGRAD\_\_REV\_\_MATRIX\_\_LOG\_SUM\_EXP\_HPP

3

4 #include <stan/agrad/rev/var.hpp>

5 #include <stan/agrad/rev/calculate\_chain.hpp>

6 #include <stan/agrad/rev/internal/vv\_vari.hpp>

7 #include <stan/agrad/rev/internal/vd\_vari.hpp>

8 #include <stan/agrad/rev/internal/dv\_vari.hpp>

9 #include <stan/agrad/rev/internal/matrix\_vari.hpp>

10 #include <stan/agrad/rev/operators/operator\_greater\_than.hpp>

11 #include <stan/agrad/rev/operators/operator\_not\_equal.hpp>

12 #include <stan/math/functions/log\_sum\_exp.hpp>

13 #include <stan/math/matrix/Eigen.hpp>

14

15 namespace stan {

16  namespace agrad {

17

18  namespace {

19

20  // these function and the following class just translate

21  // log\_sum\_exp for std::vector for Eigen::Matrix

22

23  template <int R, int C>

24  double log\_sum\_exp\_as\_double(const Eigen::Matrix<var,R,C>& x) {

25  using std::numeric\_limits;

26  using std::exp;

27  using std::log;

28  double max = -numeric\_limits<double>::infinity();

29  for (int i = 0; i < x.size(); ++i)

30  if (x(i) > max)

31  max = x(i).val();

32  double sum = 0.0;

33  for (int i = 0; i < x.size(); ++i)

34  if (x(i) != -numeric\_limits<double>::infinity())

35  sum += exp(x(i).val() - max);

36  return max + log(sum);

37  }

38

39  class log\_sum\_exp\_matrix\_vari : public op\_matrix\_vari {

40  public:

41  template <int R, int C>

42  log\_sum\_exp\_matrix\_vari(const Eigen::Matrix<var,R,C>& x) :

43  op\_matrix\_vari(log\_sum\_exp\_as\_double(x), x) {

44  }

45  void chain() {

46  for (size\_t i = 0; i < size\_; ++i) {

47  vis\_[i]->adj\_ += adj\_ \* calculate\_chain(vis\_[i]->val\_, val\_);

48  }

49  }

50  };

51  }

52

58  template <int R, int C>

59  inline var log\_sum\_exp(const Eigen::Matrix<var,R,C>& x) {

60  return var(new log\_sum\_exp\_matrix\_vari(x));

61  }

62

63  }

64 }

65 #endif

log\_sum\_exp.hpp

dv\_vari.hpp

stan::agrad::log\_sum\_exp

fvar< T > log\_sum\_exp(const fvar< T > &x1, const fvar< T > &x2)

**Definition:** log\_sum\_exp.hpp:15

operator\_greater\_than.hpp

stan::math::max

double max(const double a, const double b)

**Definition:** max.hpp:7

matrix\_vari.hpp

vd\_vari.hpp

stan::agrad::sum

fvar< T > sum(const Eigen::Matrix< fvar< T >, R, C > &m)

**Definition:** sum.hpp:14

operator\_not\_equal.hpp

var.hpp

Eigen.hpp

stan::agrad::calculate\_chain

double calculate\_chain(const double &x, const double &val)

**Definition:** calculate\_chain.hpp:8

stan::agrad::var

Independent (input) and dependent (output) variables for gradients.

**Definition:** var.hpp:27

calculate\_chain.hpp

size\_

size\_t size\_

**Definition:** dot\_self.hpp:18

vv\_vari.hpp

stan::agrad::log

fvar< T > log(const fvar< T > &x)

**Definition:** log.hpp:15

stan::agrad::exp

fvar< T > exp(const fvar< T > &x)

**Definition:** exp.hpp:16


---

[ Stan Home Page ]

*© 2011–2014,
Stan Development Team.*
